# Infective Stroke

**Published:** 2013-09

**Authors:** Somsri Wiwanitit, Viroj Wiwanitkit

**Affiliations:** 1 Wiwantikit House, Bangkhae, Bangkok, Thailand;; 2 Visiting Professor, Hainan Medical University, China; Visiting Professor, Faculty of Medicine, University of Nis, Serbia; Adjunct Professor, Joseph Ayobabalola University, Nigeria

Stroke is an important medical problem that requires good management. In medicine, stroke is the most common cause of disability around the world. The recent report on infective stroke is very interesting.^1^ Moghtaderi and Alavi-Naini^1^ pointed out many interesting issues and mentioned a large number of tropical infections that can cause infective stroke. According to this report, malaria, tuberculosis, cysticercosis, syphilis, and Chagas disease are important examples of infective stroke.^1^ In fact, several kinds of tropical infections (i.e. parasitic, bacterial, viral, or fungal infections) can lead to stroke. Moghtaderi and Alavi-Naini also noted that “Lack of human as well as financial resources makes it difficult to control and treat the disease properly.”^1^ Indeed, the epidemiology of stroke in tropical countries is interesting. Gomes and Chalela et al.^2^ noted that “Cerebrovascular disease is a leading cause of morbidity and mortality in tropical countries.” The authors also added to classical etiologies by mentioning “unusual causative mechanisms”.

Tropical infections comprise an important group that merits due consideration. It has been noted that tropical infections accumulate “up to 10% of the cases of strokes in adults” in tropical countries.^3^ The predominant tropical infections that can lead to tropical stroke might be different in different settings. For example, Chagas disease is predominant in South America, whereas gnathostomiasis is predominant in Southeast Asia.^3^ However, some tropical infections that can cause stroke such as malaria and cysticercosis can be seen in many tropical as well as non-tropical areas.^3^ Of interest, the clinical problems are sometimes underdiagnosed. For sure, this leads to the high mortality among the patients with specific tropical strokes.^3^ As evidence, del Brutto et al.^4^ mentioned that “The severity of the neurological picture makes it impossible to identify an specific stroke syndrome and cerebrovascular complications are only recognized on neuroimaging studies or autopsy.” This fact calls for medical attention to the forgotten problem of tropical stroke. Of interest, the problem of cryptogenic stroke still exists and incomplete investigation has been cited as the most important cause of it.^5^ There is no doubt that those cryptogenic cases can have the exact underlying etiologies as tropical infections.^5^ Indeed, in any setting, the differential diagnosis of stroke should include infection. It should be stressed that both tropical infectious and non-infectious diseases can be the etiologies of cerebral infarcts or hemorrhages.^4^ Therefore, the awareness of general practitioners about tropical infective stroke is the most important step toward success in the management of tropical infective stroke. At present, there are many helpful diagnostic tools in the form of both laboratory and imaging modalities. However, these tools will be rendered useless if the treating physician fails to recognize the problem.

Another point deserving of note is the possibility of disease transportation from the endemic to the non-endemic area due to enhanced transportation systems and globalization. The importance of travel medicine in infective tropical stroke must be kept in mind by all practitioners. As an example, late complications of malaria, including infective stroke, must be borne in mind in any case with a history of visiting a malaria-endemic area.^6^ Nevertheless, as was previously noted, the condition of tropical infective stroke is usually missed by the treating physician in the traveler. It has been noted that the tropical infective stroke is “challenging to diagnose unless a history of travel is elicited.”^7^ Sometimes, the problem cannot be diagnosed until the patient’s death and the autopsy reveals the exact tropical infective stroke. A good case study is the autopsy case of histoplasmosis-induced stroke in a Japanese traveler who visited several tropical endemic countries.^8^

In conclusion, it must be noted that tropical stroke can be seen in non-tropical countries. It is the role of the practitioner to pay thorough heed to this condition and conduct further diagnostic investigations with a view to establishing the final diagnosis and providing proper therapeutic management. To that end, we would make the following recommendations: a) infection should be included in the differential diagnosis of stroke; b) good history taking should not be ignored; and c) the significance of travel medicine should not be underestimated. 

## References

[1] Moghtaderi A, Alavi-Naini R (2012). Infective causes of stroke in tropical regions. Iran J Med Sci.

[2] Gomes J, Chalela JA (2005). Stroke in the tropics. Semin Neurol.

[3] Carod-Artal FJ (2007). [Strokes caused by infection in the tropics]. Rev Neurol.

[4] del Brutto OH (2001). [Cerebrovascular disease in the tropics]. Rev Neurol.

[5] Timsit S, Breuilly C (2009). [Cryptogenic cerebral infarction: from classification to concept]. Presse Med.

[6] Rothenberg G, Schubert S (1983). [Assessment of late complications of malaria in travelers to the tropics]. Z Gesamte Inn Med.

[7] Román GC (2011). The neurology of parasitic diseases and malaria. Continuum (Minneap Minn).

[8] Arai T, Fujigasaki J, Arakawa H, Nagashima H, Joki T, Murakami S (2004). [An autopsy case with cerebral histoplasmoma: case report]. No To Shinkei.

